# α-lipoic acid protects against hypoxia/reoxygenation-induced injury in human umbilical vein endothelial cells through suppression of apoptosis and autophagy

**DOI:** 10.3892/mmr.2015.3351

**Published:** 2015-02-13

**Authors:** JINGJING ZHANG, HOULIANG DENG, LI LIU, XIAOXIA LIU, XIALIN ZUO, QIAN XU, ZHUOMIN WU, XIAOBIN PENG, AIMIN JI

**Affiliations:** 1 Center for Drug Research and Development, Zhujiang Hospital, Southern Medical University, Guangzhou, Guangdong 510282, P.R. China; 2 Institute of Neurosciences, The Second Affiliated Hospital of Guangzhou Medical University, Guangzhou, Guangdong 510260, P.R. China; 3 School of Pharmaceutical Sciences, Southern Medical University, Guangzhou, Guangdong 510515, P.R. China

**Keywords:** human umbilical vein endothelial cells, hypoxia/reoxygenation, α-lipoic acid, apoptosis, autophagy

## Abstract

α-lipoic acid (ALA) is known as a powerful antioxidant, which has been reported to have protective effects against various cardiovascular diseases. The present study aimed to determine whether ALA pre- or post-treatment induced protective effects against hypoxia/reoxygenation-induced injury via inhibition of apoptosis and autophagy in human umbilical vein endothelial cells (HUVECs). In order to simulate the conditions of hypoxia/reoxygenation, HUVECs were subjected to 4 h of oxygen-glucose deprivation (OGD) followed by 12 h of reoxygenation. For the pre-treatment, ALA was added to the buffer 12 h prior to OGD, whereas for the post-treatment, ALA was added at the initiation of reoxygenation. The results demonstrated that ALA pre- or post-treatment significantly reduced lactate dehydrogenase (LDH) release induced through hypoxia/reoxygenation in HUVECs in a dose-dependent manner; of note, 1 mM ALA pre- or post-treatment exhibited the most potent protective effects. In addition, ALA significantly reduced hypoxia/reoxygenation-induced loss of mitochondrial membrane potential, apoptosis and the expression of cleaved caspase-3 in HUVECs. In the presence of the specific autophagy inhibitor 3-methyladenine, hypoxia/reoxygenation-induced apoptosis was significantly reduced. Furthermore, the formation of autophagosomes, cytosolic microtubule-associated protein 1A/1B-light chain 3 ratio and beclin1 levels significantly increased following hypoxia/reoxygenation injury; however, all of these effects were ameliorated following pre- or post-treatment with ALA. The results of the present study suggested that ALA may provide beneficial protection against hypoxia/reoxygenation-induced injury via attenuation of apoptosis and autophagy in HUVECs.

## Introduction

Cardiovascular complications are the leading cause of morbidity and mortality in the United States (1). Oxidative stress, a main risk factor of vascular endothelial cell injury, is known to be involved in the pathogenesis of cardiovascular disorders (2). Oxidative stress induces apoptosis in various types of cancer cell (3) and participates in the regulation of autophagy under certain circumstances (4). Apoptosis and autophagy are two major forms of programmed cell death (5). Apoptosis is characterized by obvious morphological features, including cell membrane blebbing, cellular volume reduction, caspase activation, chromatin condensation and nuclear fragmentation (6), whereas autophagy is a catabolic process, which enables cells to recycle amino acids and other intracellular nutrients, allowing them to obtain energy from recycled materials (7). Excessive autophagic activity results in the total collapse of all cellular functions, resulting in the loss of large amount of the cytosol and organelles (8), which can lead to autophagic cell death. There is a complex association between apoptosis and autophagy depending on cell types and the category of the stimulus (9). Under certain conditions, autophagy and apoptosis appear to display positive and negative interactions (10); however, they can also coexist or occur sequentially in numerous circumstances (11).

α-Lipoic acid (ALA) is an endogenous short-chain fatty acid. ALA and its reduced form dihydrogen lipoic acid (DHLA) are known to act as antioxidants *in vitro* and *in vivo*; in addition, ALA has been reported to reduce oxidative stress (12) and apoptosis (13). Previous studies have indicated that the beneficial effect of ALA was associated with autophagy (14,15).

Vascular endothelial cells (EC) are important for maintaining vasculature integrity throughout the body (16). Endothelial damage is the initial event in cardiovascular disease; however, to the best of our knowledge, there have been limited comprehensive studies performed in order to investigate whether ALA exhibits protective effects against hypoxia/reoxygenation-induced injury in human umbilical vein endothelial cells (HUVECs). The aim of the present study was to examine whether ALA pre- or post-treatment induced protective effects against hypoxia/reoxygenation-induced injury in HUVECs. Furthermore, the present study aimed to determine whether ALA-induced beneficial effects in HUVECs were associated with apoptosis and autophagy.

## Materials and methods

### HUVEC exposure to oxygen-glucose deprivation (OGD) and ALA treatment

HUVECs were obtained from the American Type Culture Collection (Manassas, VA, USA) and cultured in Dulbecco’s modified Eagle’s medium (DMEM; Gibco, Invitrogen Life Technologies, Carlsbad, CA, USA) supplemented with 10% fetal bovine serum (FBS; Gibco) in 100 U/ml penicillin and 100 *μ*g/ml streptomycin (Gibco) with 5% CO_2_ atmosphere at 37°C. HUVECs were subjected to OGD by washing twice with phosphate-buffered saline (PBS; Shanghai Bogoo Biological Tecnology Co., Ltd, Shanghai, China), replacing the medium with glucose-free DMEM and then transferring the plates into an anaerobic chamber (Billups-Rothenberg Inc., Del Mar, CA, USA), filled with 100% N_2_ (Foshan MS Messer Gas Co., Ltd, Foshan, China) for 15 min and sealed at 37°C for 4 h. The medium was then replaced with high-glucose DMEM and the plates were returned to a 5% CO_2_/95% air incubator for reoxygenation. ALA stock solution (Shanghai Modern Pharmaceutical Co., Ltd, Shanghai, China) was prepared with minimal exposure to air and light, then stored at 4°C. For ALA pre-treatment, various concentrations of ALA (0.25, 0.5, 1 and 2 mM) were added to the culture medium and incubated for 12 h prior to OGD. For ALA post-treatment, ALA was added at the beginning of reoxygenation. The control group were subjected to identical experimental procedures without ALA treatment. For the treatment with autophagy inhibitor, cells were pre-incubated with 1 mM 3-methyladenine (3-MA; Sigma-Aldrich, St. Louis, MO, USA) for 1 h prior to OGD. Further steps were identical to those in the control group.

### Lactate dehydrogenase (LDH) assay

LDH activity in the supernatant cell culture media was measured using a commercially available kit (Cytotoxicity Detection LDH kits; Nanjing Jiancheng Chemical Industrial Co., Ltd, Nanjing, China), according to the manufacturer’s instructions.

### Flow cytometric analysis of apoptosis

Cell apoptosis was detected using an Annexin V-fluorescein isothiocyanate (FITC)/propidium iodide (PI) kit (BD Biosciences, San Jose, CA, USA). In brief, cells were collected, washed twice with ice-cold PBS and then suspended in 300 *μ*l binding buffer containing 5 ml Annexin V and 5 ml PI in the dark for 15 min at room temperature. The cell suspension was immediately analyzed using flow cytometry (FACSCalibur; BD Biosciences, Franklin Lakes, NJ, USA). Each group (1×10^−4^ cells) was examined and the percentages of viable (AV−, PI−), apoptotic (AV+,PI−), apoptotic and necrotic (AV+,PI+) and cells that were already dead (AV−,PI+) cells were analyzed.

### Mitochondrial membrane potential assay (Δψ_m_)

Δψ_m_ was evaluated using fluorescence microscopy with Rho123 (Sigma, St. Louis, MO, USA) as the fluorescent probe. Cells were rinsed with PBS and then stained with Rho123 (0.005 mg/ml) for 20 min at 37°C. Following washing twice with PBS, samples were measured under a fluorescence microscope (AF6000; Leica Microsystems, Wetzlar, Germany) at an excitation wavelength of 488 nm and an emission wavelength of 535 nm.

### Transmission electron microscopy (TEM)

Cells (2×10^5^) were seeded in 6-cm dishes and incubated under hypoxia/reoxygenation with or without ALA or normoxic conditions. HUVECs in different groups were collected and then centrifuged at 1,120 × g for 5 min. The cell pellets were then fixed with 4% paraformaldehyde (Shanghai Majorbio Bio-Pharm Technology Co., Ltd, Shanghai, China) and 2.5% glutaralde-hyde (Shanghai Majorbio Bio-Pharm Technology Co., Ltd,) and stored at 4°C prior to TEM in the electron microscope room of Southern Medical University, Guangzhou, China. Images were obtained using an electron microscope (EC UC7; Leica Microsystems).

### Monodansylcadaverine (MDC) staining

ALA-treated and control HUVECs were stained using MDC (Sigma-Aldrich) in order to detect autophagosomes. HUVECs were washed with PBS, then stained with 0.05 mM MDC at 37°C for 10 min. Following incubation, cells were washed twice with PBS and the samples were analyzed under a fluorescence microscope.

### Western blot analysis

HUVECs were grown on 100-mm plates subjected to hypoxia/reoxygenation in the presence or absence of ALA. Western blot analysis was performed as follows: Cells were washed with PBS, lysed in ice-cold radioimmunoprecipitation assay buffer (Nanjing Keygen Biotech Co., Ltd, Nanjing, China) containing 1% protease inhibitor cocktail (Nanjing Keygen Biotech Co., Ltd) for 30 min prior to centrifugation at 13,000 × g for 10 min at 4°C. The protein concentration was quantified using a bicinchoninic acid assay kit (Pierce Biotechnology, Inc., Rockford, IL, USA) and measured by a microplate reader (model 680, Bio-Rad, Rutland, VT, USA). Proteins were separated using 8–10% SDS-PAGE and transferred to polyvinylidene fluoride membranes (EMD Millipore, Bedford, MA, USA). The membranes were blocked for 3 h at room temperature in 5% skimmed milk (Wuhan Boster Bio-Engineering Co., Ltd, Wuhan, China), then incubated overnight at 4°C with primary antibodies, including rabbit anti-rat polyclonal LC3, rabbit anti-rat polyclonal beclin1 and rabbit anti-rat polyclonal cleaved caspase-3 (Cell Signaling Technology, Inc., Danvers, MA, USA). The membranes were then incubated with rabbit secondary antibodies (1:5,000; Wuhan Boster Bio-Engineering Co., Ltd) for 1 h at room temperature. Rabbit anti-rat monoclonal β-tubulin and rabbit anti-rat monoclonal GAPDH (1:1,000; Cell Signaling Technology, Inc., Danvers, MA, USA) were used as the internal controls. Protein expression was identified using an enhanced chemiluminescence advance western blot detection kit (Pierce Biotechnology, Inc.).

### Statistical analysis

Experiments were performed at least in triplicate and results were analyzed using SPSS 13.0 software (SPSS, Inc., Chicago, IL, USA). Values are expressed as the mean ± standard deviation. Differences of means among groups were assessed using a one way analysis of variance. P<0.05 was considered to indicate a statistically significant difference between values.

## Results

### ALA pre- or post-treatment induces protective effects against hypoxia/reoxygenation-induced injury in HUVECs

**Figure 1:**
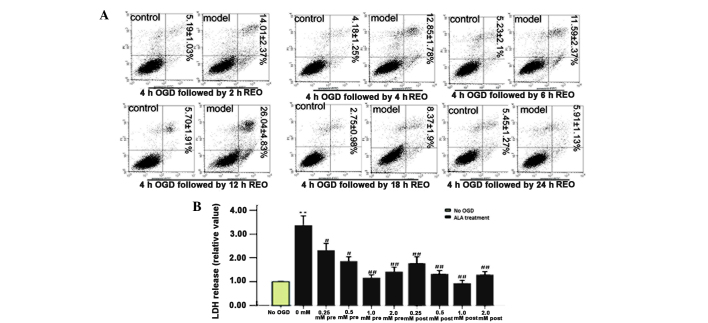
ALA pre- and post-treatment induces protective effects in HUVECs. (A) Quantification assay for apoptotic activity was evaluated using flow cytometry. HUVECs were exposed to OGD for 4 h followed by reoxygenation for different time periods, with 12 h of reoxygenation inducing the highest levels of apoptosis. Percentages of apoptotic cells (lower right quadrant) as well as apoptotic and necrotic cells (upper right quadrant) are presented as the mean ± standard deviation (n=3). (B) Effect of ALA on LDH release. HUVECs were exposed to OGD for 4 h followed by 12 h of reoxygenation. Pre- or post-treatment with ALA at various concentrations (0.25–2 mM) decreased the OGD-induced increase in LDH release in a concentration-dependent manner. Values are presented as the mean ± standard deviation. ^**^P<0.01 vs. no OGD; ^#^P<0.05 and ^##^P<0.01 vs. 0 mM ALA treatment. HUVECs, human umbilical vein endothelial cells; OGD, oxygen-glucose deprivation; LDH, lactate dehydrogenase; ALA, α-lipoic acid; REO, reoxygenation.

The extent of cell damage, apoptosis and necrosis was found to increase with the duration of OGD (Fig. 1A). Therefore, a duration of 4 h of OGD followed by 12 h of reoxygenation (apoptotic rate ~25%) was selected as the OGD model for subsequent experiments.

LDH release was measured in order to investigate whether ALA pre- or post-treatment induced protective effects against hypoxia/reoxygenation-induced cell injury and determine the appropriate concentration of ALA. The results showed that ALA exhibited protective effects against hypoxia/reoxygenation-induced injury with pre- as well as post-treatment in a concentration-dependent manner (Fig. 1B). In addition, the hypoxia/reoxygenation-induced LDH activity was significantly reduced following pre- or post-treatment at all concentrations of ALA; however, 1 mM ALA was found to be the most effective concentration and was therefore used for all subsequent experiments.

### Hypoxia/reoxygenation-induced apoptosis and autophagy in HUVECs

**Figure 2:**
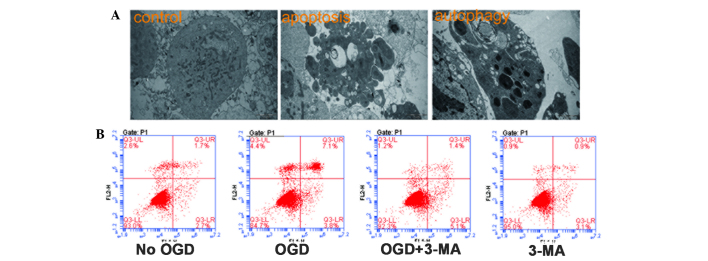
Hypoxia-induced apoptosis and autophagy in HUVECs. (A) HUVECs were treated with OGD for 4 h followed by 12 h reoxygenation. Apoptosis and autophagy were then observed using electron microscopy (magnification, ×12,000). (B) HUVECs were treated with 3-MA and apoptosis was measured using flow cytometry. Early apoptotic or apoptotic and necrotic cells were identified as single positive for FITC-Annexin V (lower right quadrant) or double positive for both FITC-Annexin V and propidium iodide (upper right quadrant), respectively. HUVECs, human umbilical vein endothelial cells; OGD, oxygen-glucose deprivation; FITC, fluorescein isothiocyanate; 3-MA, 3-methyladenine.

HUVECs were exposed to 4 h of hypoxia followed by 12 h of reoxygenation and TEM was used to observe cell apoptosis and autophagy (Fig. 2A). In addition, the specific autophagy inhibitor 3-methyladenine (MA) was employed in order to investigate the association between apoptosis and autophagy (Fig. 2B). The results demonstrated that treatment with 3-MA markedly decreased the apoptotic rate compared with that of the OGD group (~6.5 and 11.0%, respectively) (Fig. 2B). These results therefore indicated that hypoxia/reoxygenation-induced apoptosis as well as autophagy in HUVECs and that the inhibition of autophagy contributed to the downregulation of apoptosis.

### ALA pre- or post-treatment reduces hypoxia/reoxygenation-induced apoptosis in HUVECs

**Figure 3:**
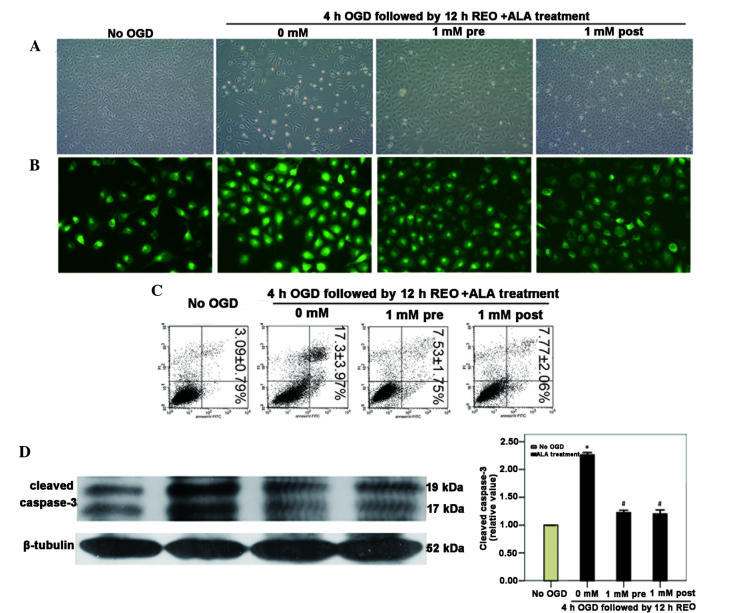
ALA pre- or post-treatment reduces HUVEC apoptosis induced by OGD/reoxygenation. HUVECs were subjected to 4 h of OGD followed by 12 h of reoxygenation in the presence or absence of 1 mM ALA pre- or post-treatment. (A) Cell morphology was observed using inverted phase contrast microscopy (magnification, ×100). (B) Fluorescence microscopy with Rho123 staining was used to detect the mitochondrial membrane potential (magnification, ×400). (C) Cell apoptosis was measured using flow cytometry. Percentages of apoptotic cells (lower right quadrant) as well as apoptotic and necrotic cells (upper right quadrant) are presented as the mean ± standard deviation (n=3). (D) Western blot analysis was used to measure cleaved caspase-3 expression levels and quantitative analysis of these western blots revealed that cleaved caspase-3 was significantly downregulated in ALA pre- or post-treatment groups. ^*^P<0.05 vs. no OGD; ^#^P<0.05 vs. 0 mM ALA treatment. HUVECs, human umbilical vein endothelial cells; OGD, oxygen-glucose deprivation; ALA, α-lipoic acid; REO, reoxygenation.

In order to determine whether ALA induced pre- or post-protective effect against hypoxia/reoxygenation-induced apoptosis in HUVECs, cell morphology was observed using inverted phase contrast microscopy (Fig. 3A). The association between apoptosis and the disruption of Δψ_m_ is well documented (17). Therefore, in the present study, Δψ_m_ was detected using Rho123, where the intensity of fluorescent staining reflected the loss of the Δψ_m_. The results showed that ALA attenuated the loss of Δψ_m_, which was induced by hypoxia/reoxygenation (Fig. 3B).

Apoptosis of HUVECs was detected using flow cytometry, and the protein expression of cleaved caspase-3 was detected using western blot analysis. The flow cytometry results demonstrated that ALA pre- or post-treatment significantly reduced apoptosis compared with that of the OGD-untreated group (Fig. 3C). In concurrence with these results, western blot analysis revealed that cleaved caspase-3 expression levels were significantly increased following OGD; however, 1 mM ALA markedly reduced cleaved caspase-3 expression levels compared with those in the untreated group (Fig. 3D).

### ALA pre- or post-treatment reduces hypoxia/reoxygenation-induced autophagy in HUVECs

**Figure 4:**
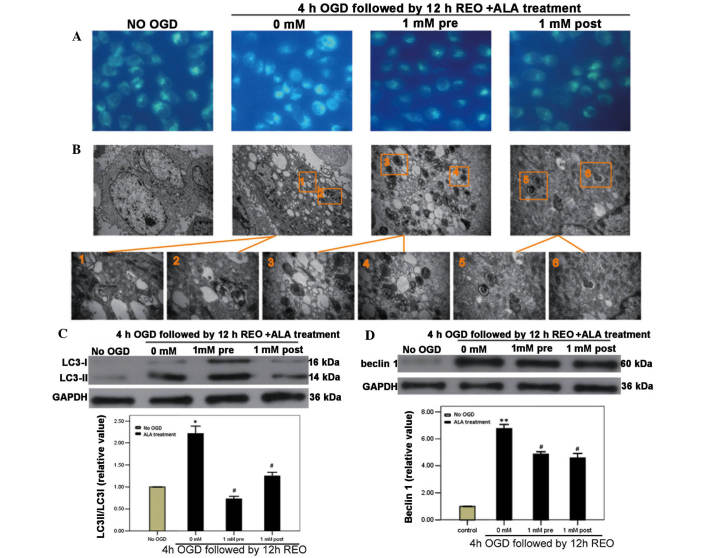
ALA pre- or post-treatment reduces hypoxia/reoxygenation-induced autophagy in HUVECs. HUVECs were subjected to 4 h of OGD followed by 12 h reoxygenation in the presence or absence of 1 mM ALA pre- or post-treatment. (A) Autophagic vacuoles were stained with monodansylcadaverine, which was detected using fluorescence microscopy (magnification, ×500). (B) Autophagic vacuoles were observed using an electron microscope (magnification upper, 12,000x; lower, 60,000x). Western blot analysis revealed that (C) the conversion of LC3-I to LC3-II was significantly increased in the OGD group and (D) beclin1 was significantly increased in the OGD group, whereas ALA pre- or post-treatment attenuated the effects of hypoxia/reoxygenation-induced injury. ^*^P<0.05 and ^**^P<0.01 vs. no OGD; ^#^P<0.05 vs. 0 mM ALA treatment. HUVECs, human umbilical vein endothelial cells; OGD, oxygen-glucose deprivation; ALA, α-lipoic acid; REO, reoxygenation.

In order to determine whether ALA induced pre- or post-protective effects against hypoxia/reoxygenation-induced autophagy in HUVECs, autophagic vacuoles were observed using MDC staining and TEM; in addition, the expression of the autophagic marker proteins LC3B and beclin1 was detected using western blot analysis. The results demonstrated that ALA pre- or post-treatment significantly decreased the number of MDC-labeled fluorescent particles and autophagic vacuoles compared with those of the OGD untreated group (Fig. 4A and B). Furthermore, ALA pre- and post-treatment significantly downregulated the expression of beclin1 and the conversion of LC3-I to LC3-II compared with those in the OGD untreated group (Fig. 4C).

## Discussion

Endothelial cells have key roles in the physiological and pathophysiological regulation of the cardiovascular system; therefore, endothelial dysfunction is an important risk factor for the development of clinical events, including vascular diseases and stroke (18). Furthermore, endothelial cell injury is the initial step of cardiovascular disease, which may lead to vasospasms, blood clots, atheromatous plaque formation as well as injury to the parenchymal cells and organs (19). Therefore, the effective protection of endothelial cells is essential for the prevention and treatment of cardiovascular injuries. ALA has been previously reported to exert beneficial effects on various diseases associated with vascular dysfunction (20). In addition, ALA was shown to have protective effects against the ischemia/reperfusion-induced injury in several tissues, including renal (21), retinal (22) and myocardial tissues (23). In the present study, concentration-dependent pre- and post-protective effects of ALA were observed against hypoxia/reoxygenation-induced cell injury in HUVECs, which occurred via the suppression of apoptosis and autophagy.

LDH activity is a common marker of cell viability which is used to assess endothelial injury (24). The results of the present study were consistent with another report on the beneficial effects of ALA against ischemia, which reported that ALA reduced LDH activity in cerebral endothelial cells (25). The present study demonstrated that ALA pre- or post-treatment exerted a concentration-dependent protective effect against hypoxia/reoxygenation-induced injury in HUVECs.

Mitochondrial oxidative stress has a central role in the intrinsic pathway, which results in loss of Δψ_m_ and caspase activation. Activation of caspases is important for the initiation and completion of the apoptotic processes (26). The cleavage of caspase-3, a marker protein of apoptosis, is the final step of the extrinsic and intrinsic apoptotic pathways (27). The results of the present study revealed that ALA pre- or post-treatment prevented the collapse of Δψ_m_, reduced the expression of cleaved caspase-3 and inhibited cell apoptosis compared with the untreated HUVECs following hypoxia/reoxygenation-induced injury.

A limited number of studies have focused on the role of autophagy in oxidative stress. One previous study reported that pre-treatment with ALA inhibited autophagy in H9c2 cells exposed to hypoxia/reoxygenation (28). However, the association between apoptosis and autophagy with the differential effect of ALA pre- and post-treatments has not yet been investigated. The ratio of cytosolic LC3-II/LC3-I is a sensitive and quantitative index of autophagic flux, which differs from that of a dominant active form of LC3-II in the membrane fraction (29). The results of the present study showed that ALA decreased LC3 conversion, therefore indicating that ALA was able to suppress autophagy; these results were consistent with those reported by Karim *et al* (15). Beclin1 is also an important protein involved in the onset of autophagy, which controls the levels of p53 (30). Beclin1 interacts with anti-apoptotic multi-domain proteins of the B cell lymphoma 2 family; disruption of these interactions may liberate beclin1 proteins, which may result in the activation of autophagy (31). Therefore, autophagy is regarded as a pro-apoptotic factor and the cause of ‘type II’ programmed cell death (32). The results of the present study demonstrated that beclin1 expression was significantly increased following hypoxia/reoxygenation-induced injury in HUVECs, whereas ALA was found to attenuate this increase.

In conclusion, the results of the present study demonstrated that pre- or post-treatment with ALA resulted in the reduction of LDH activity in HUVECs. In addition, ALA was found to exert its protective effects via the suppression of mitochondrial- and caspase-dependent apoptosis as well as autophagy, which were rapidly upregulated in HUVECs exposed to hypoxia/reoxygenation.
